# Effect of Equal Channel Angular Pressing on the Dynamic Softening Behavior of Ti-6Al-4V Alloy in the Hot Deformation Process

**DOI:** 10.3390/ma14010232

**Published:** 2021-01-05

**Authors:** Zhiyong Zhao, Jun Gao, Yaoqi Wang, Yanling Zhang, Hongliang Hou

**Affiliations:** 1 School of Mechanical, Electrical and Information Engineering, Shandong University (Weihai), Weihai 264209, China; 2 AVIC Manufacturing Technology Institute, Beijing 100024, China; zzymhylove@163.com (Y.W.); zhangyanling205@163.com (Y.Z.); houhongl@163.com (H.H.)

**Keywords:** titanium alloy, equal channel angular pressing, hot deformation behavior, dynamic softening

## Abstract

To investigate the effect of equal channel angular pressing (ECAP) on the deformation of Ti-6Al-4V alloy at a higher temperature, hot compression tests were conducted on alloys having two different initial microstructures (the original alloy (Pre-ECAP) and ECAP-deformed alloy (Post-ECAP)). Post-ECAP, the alloy showed a higher degree of dynamic softening during the hot deformation process due to its finer grain size and higher distortion energy. The flow stress of Post-ECAP alloy was higher than the Pre-ECAP alloy at 500 °C when $\dot{\varepsilon }=\;0.003{\;s}^{-1}$. However, the stress of the Post-ECAP alloy decreased rapidly with increasing temperature and strain rate, until the stress value was much lower than that of Pre-ECAP at 700 °C when $\dot{\varepsilon }=\;0.03\;s^{-1}$. The value of the dynamic softening coefficient revealed that the dynamic softening behavior of Post-ECAP was more pronounced than that of Pre-ECAP in the hot compression deformation process. The main dynamic softening mechanism of Pre-ECAP is dynamic recovery, while the dynamic recrystallization process plays a more important role in the deformation process of Post-ECAP alloy. The microstructures observation results showed that dynamic recrystallization was more likely to occur to Post-ECAP alloys under the same deformation condition. Almost fully dynamic recrystallization had occurred in the deformation process of Post-ECAP at 700 °C and a strain rate of $\dot{\varepsilon }=\;0.01\;s^{-1}$. The grains of Post-ECAP alloys were further refined. The Post-ECAP alloy exhibits better plastic deformation at temperatures higher than 600 °C due to its significant dynamic recrystallization.

## 1. Introduction

Titanium and its alloys have been widely used in astronautics, aeronautics, and marine fields due to their high strength-to-weight ratios, excellent fracture toughness, and good resistance to corrosion/oxidation [1,2]. Ti-6Al-4V alloy is regarded as a typical two-phase (α and β) alloy possessing stable microstructures and mechanical properties, which is widely used for aerospace and biomedical applications [3,4,5,6,7]. However, the difficulty in fabricating and processing the materials in the ambient or low-temperature conditions is the major obstacle that limits the widespread applications of the titanium alloys [8,9,10]. Thermoforming and superplastic forming techniques are the two major technologies for producing titanium alloys [11,12,13]. The superplastic forming technology is primarily used for making the complex shape parts, such as a multi-layer structure and irregularly curved surface. The superplastic forming technique for fabricating titanium alloy is usually carried out at high temperatures and low strain rates. The superplastic forming technique has low efficiency and is cost-ineffective. The simple titanium parts are usually processed using the thermoforming technique at temperatures between 600 and 800 °C (lower than superplastic forming) [14]. Studying the deformation behavior during the thermoforming process of fabricating the titanium alloy is important for optimizing the technology parameters.

Microstructure plays a dominant role in the hot deformation process of titanium alloy and greatly affects the dynamic softening mechanism. Zhang et al. investigated the hot deformation behavior of the Ti-6Al-4V alloy with α’ martensitic microstructure, transformed β microstructure, and bimodal microstructure between 800 and 850 °C [15]. The results revealed that dynamic recrystallization was a more likely process for alloys with α’ martensitic microstructures and bimodal microstructure. The dynamic globularization of lamellar α structure occurred only at low strain rates. The α’ martensitic starting microstructure helps achieve grain refinement in the Ti-6Al-4V alloy during the hot deformation process. Luo et al. studied the effects of the α grain size on the deformation behavior of Ti-6Al-4V during hot compression process with temperature range of 860–960 °C [16]. The results show that the flow stress increases with increasing α grain size but decreases with increasing equiaxed α phase. Ti-6Al-4V alloy with smaller grains exhibits a strong grain-boundary sliding mode along with a dominant dislocation glide/climb mechanism during hot deformation at lower temperatures and higher strain rate. The stress decreased with increasing temperature and decreasing strain rate due to the significant dynamic softening behavior. However, decreasing temperature and increasing strain rate, the two important factors in titanium alloy deformation technology, can be achieved by improving the alloy’s microstructure.

The ultra-fine-grain (UFG) materials have always been considered as perfect materials in terms of strength and plastic deformation capacity [17,18,19]. The UFG Ti-6Al-4V alloy has numerous advantages, such as high strength, good plastic deformability, and superplasticity at low temperatures and high strain rate [20,21,22]. However, the preparation of the UFG Ti-6Al-4V alloy remains a challenge. Severe plastic deformation (SPD) technology is regarded as one of the most efficient methods to fabricate UFG materials, which introduce an ultra-large plastic strain into a bulk metal during forming processes [23,24,25]. Equal channel angular pressing (ECAP) was proposed by Segal in 1977, which is an effective SPD technology [26] and has been proven to prepare UFG Ti-6Al-4V alloy. However, the ECAP process is difficult due to high strength and poor plastic deformability at low temperatures (<0.5*T_m_*) of Ti-6Al-4V alloy. Moreover, the ECAP-deformed Ti-6Al-4V alloy usually exhibits high strength and low ductility at room temperature due to its high dislocation density, work hardening, and unstable microstructure [27,28]. Furthermore, reports on the hot deformation behavior of ECAP-deformed Ti-6Al-4V alloy is scarce. The objective of the present study is to investigate the effect of ECAP on the hot deformation behavior of Ti-6Al-4V alloy. The hot compression tests were conducted at different temperatures and strain rates, and analysis of experimental data and microstructure revealed the dynamic softening behavior.

## 2. Experimental Procedure

**Figure 1:**
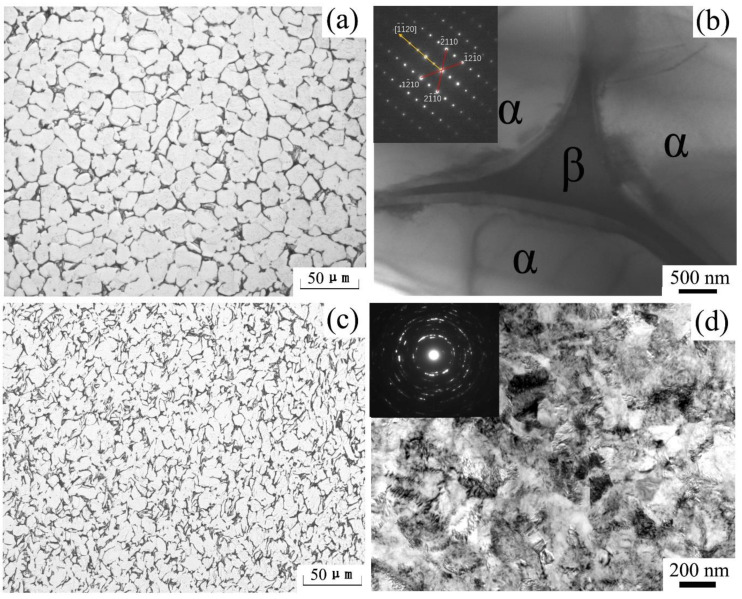
Microstructure of original alloy prior to equal channel angular pressing (Pre-ECAP) and ECAP-deformed alloy (Post-ECAP). (**a**) Optical microscopy (OM) microstructure of Pre-ECAP; (**b**) TEM microstructure of Pre-ECAP; (**c**) OM microstructure of Post-ECAP; (**d**) TEM microstructure of Post-ECAP.

A Ti-6Al-4V alloy bar (chemical component: 6.66% Al, 5.13% V, 0.21% Fe, 0.03% Mo, balance Ti) was selected as the experimental material and subjected to heat for controlling the microstructure. The equiaxed microstructure with an average grain size of ≈30 µm was obtained upon heating (at 950 °C for 2 h) and subsequent furnace cooling (Figure 1a,b). The annealed Ti-6Al-4V alloy (Pre-ECAP) consisted of a majority of equiaxed hexagonal (HCP) α-phase and a finely dispersed cubic (BCC) β-phase between α grains, which were selected as the ECAP materials.

**Figure 2:**
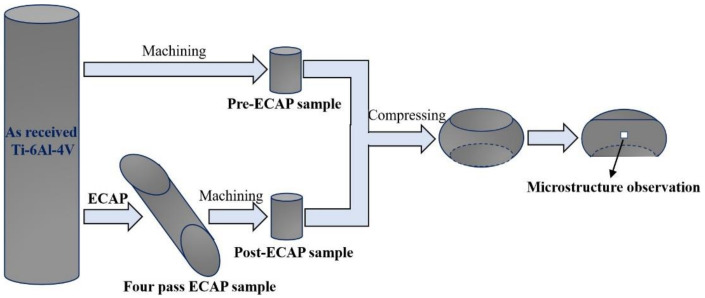
Schematic representation of experiments.

The experimental procedure is presented in Figure 2. The ECAP process of Ti–6Al–4V alloy was presented in our previous study [29]. The average grain size of Ti–6Al–4V alloy decreased to about 10 µm after a four-pass ECAP procedure (Figure 1c). However, smaller structures can be seen in the TEM images of the four-pass ECAP sample (Figure 1d). The grain size of Ti-6Al-4V alloy decreased to hundreds of nanometers, and the unclearly defined grain boundaries indicated that the microstructure was unstable due to the high dislocation density. The corresponding ringed selected area diffraction (SAD) patterns with diffused spots indicated that the misorientation of boundaries was fairly significant.

Pre-ECAP and Post-ECAP compression samples with a 6 mm diameter and a height of 9 mm were machined from the annealed Ti-6Al-4V bar and ECAP-deformed samples, respectively. Isothermal compression tests were conducted using a Gleeble-3800 simulator in the temperature range of 500–700 °C under constant true strain rates of 0.003, 0.01, and 0.03 s^−1^, respectively. The experimental compression conditions are displayed in Table 1. The samples were heated to the deformation temperatures at a heating rate of 10 °C·s^−1^, held for 5 min to homogenize the temperature throughout the specimens, and then pressed to reduce the height by 50% under a constant strain rate condition. The procedure was followed by water cooling.

Chemical attack for the samples with 30 mL HF + 10 mL HNO_3_ + 30 mL H_2_O was conducted, and the optical microscopy (OM, LEICA DMI5000) technique was used to observe the microstructures. Transmission electron microscopy (TEM) experiments were performed using a JEM-2100(H) microscope operated under voltage of 200 kV. Thin foils, which were sectioned parallel to the flow plane, were twin-jet electropolished with a solution of 20 vol.% H_2_SO_4_ in methanol before the experiments. Electron back-scattered diffraction (EBSD) experiments were conducted to examine the microstructure evolution of Ti-6Al-4V alloy, which was performed on a ZEISS-EVO18 scanning electron microscope (SEM) with a scanning step size of 0.08 µm, and HKL Channel 5 software was used to analyze the results. All the specimens for the microstructure observation were prepared parallel to the extrusion direction. All microstructures were observed at the center of the specimen, as shown in Figure 2. Samples were prepared following the standard metallographic grinding and polishing procedure and finished with 1 µm diamond suspension followed by electropolishing with perchloric acid (10%).

## 3. Results

### 3.1. Stress–Strain Behavior

**Figure 3:**
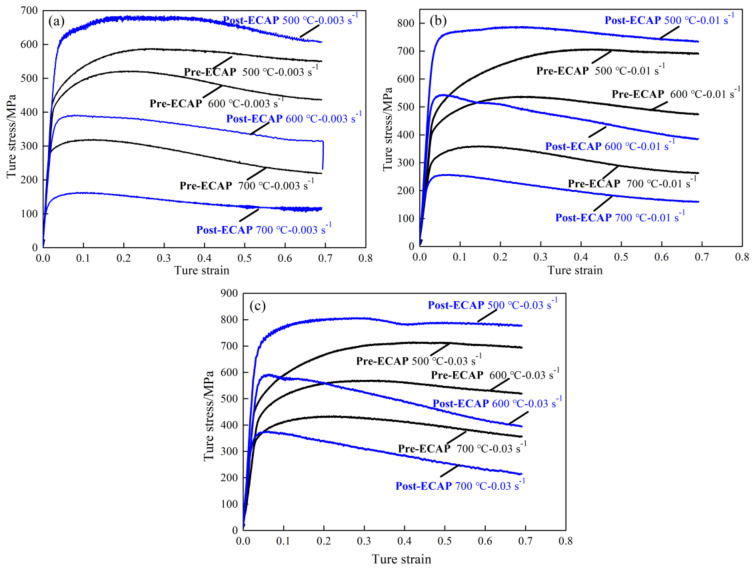
True stress-true strain curves of Ti-6Al-4V alloy in hot compression tests: (**a**) $\dot{\varepsilon }=0.003\;s^{-1}$; (**b**) $\dot{\varepsilon }=0.01\;s^{-1}$; (**c**) $\dot{\varepsilon }=0.03\;s^{-1}$.

True stress–true strain curves of Pre-ECAP and Post-ECAP at the temperatures of 500, 600, and 700 °C and strain rates of 0.003, 0.01, and 0.03 s^−1^ are summarized in Figure 3. The true stress of Pre-ECAP and Post-ECAP both increase with increasing strain rate and decrease with the increase of temperature. However, the Pre-ECAP and Post-ECAP samples have different characteristics with increasing strain rates at different temperatures. The stress of Post-ECAP is higher than Pre-ECAP under all strain rates at 500 °C. The stress of Pre-ECAP increased with increasing strain beyond the yield point, but the change in the stress of Post-ECAP is insignificant. When the deformation temperature increases to 600 °C, the stress of the Post-ECAP sample is higher than the Pre-ECAP sample at a strain rate of 0.003 s^−1^. Although the yield stress of Post-ECAP is higher than that of Pre-ECAP at a strain rate of 0.01 s^−1^ and 0.03 s^−1^, the flow stress of Post-ECAP is lower than that of Pre-ECAP with increasing strain. The stress of Pre-ECAP slightly decreased, but that of Post-ECAP decreased rapidly with increasing strain. When the deformation temperature rose to 700 °C, the stress of Post-ECAP is lower than Pre-ECAP at all strain rates, and the stress decreasing rate of Post-ECAP is higher than Pre-ECAP with increasing strain. The hot compression results indicate that the variation of temperatures and strain rates have a larger effect on the deformation behavior of Post-ECAP than that of Pre-ECAP.

### 3.2. Effect of Temperature and Strain Rate on Deformation Behavior of Ti-6Al-4V Alloy

**Figure 4:**
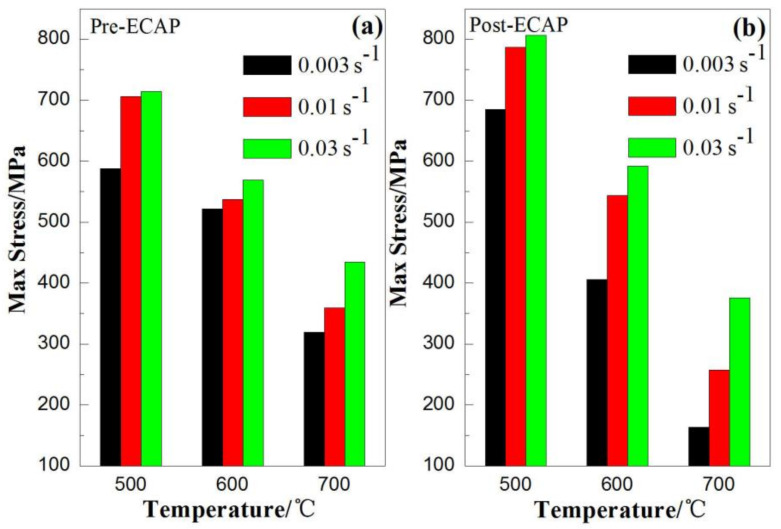
Maximum stress variation in the Ti-6Al-4V alloy with temperature and strain rate for (**a**) Pre-ECAP; (**b**) Post-ECAP samples.

Microstructure, temperature, and strain rate are the three major factors that influence the deformation behavior of metal materials. The differences in hot deformation behavior at various temperatures and strain rates are caused by different initial microstructure in the Pre-ECAP and Post-ECAP. Figure 4 shows the maximum stress variation of the Ti-6Al-4V alloy with temperature and strain rate. It can be seen from Figure 4 that the maximum stress of Post-ECAP was sensitive to both temperature and strain rate; i.e., at the same temperature, the change in the value of the maximum stress with strain rate for Post-ECAP is greater than that for Pre-ECAP. At the same strain rate, the change in the value of the maximum stress with temperature for the Post- ECAP is also greater than that of Pre-ECAP.

As Figure 1 shows, the grain size of the Post-ECAP samples is smaller than the Pre-ECAP samples. Dislocations and deformation twins were induced during the ECAP process [29,30], which lead to dynamic recovery and dynamic recrystallization during the hot compression deformation process. Dynamic recovery is the main softening mechanism at 500 °C, as it is difficult to induce dynamic recrystallization at such a low temperature. As a result, the higher dislocation density in Post-ECAP samples leads to higher stress, making deformation difficult. As the deformation temperature increases, the dynamic softening characteristics of the Post-ECAP samples’ stress–strain curves become significant. This is because the dynamic softening and dynamic recrystallization techniques were promoted by the increasing mobile dislocations and slip systems under low strain rate conditions. As the grains of the Post-ECAP samples are smaller than the Pre-ECAP samples, the diffusion creep effect can play a significant role in the deformation process and provide enough time for dynamic softening behavior leading to significantly decreased flow stress [31]. Therefore, the flow stress of the Post-ECAP is lower than the Pre-ECAP samples at a temperature of 600 °C and strain rate of $\dot{\varepsilon }=0.003{\;s}^{-1}$. However, the yield stress of the Post-ECAP is still higher than the Pre-ECAP sample at a high strain rate due to inadequate dynamic softening. The similar case was observed with the deformations at 700 °C and strain rate at $\dot{\varepsilon }=\;0.003{\;s}^{-1}$. The peak stress of the Post-ECAP sample is far less than the Pre-ECAP sample, with the corresponding value of 160 and 320 MPa, respectively. However, when the strain rate increases to $\dot{\varepsilon }=\;0.03{\;s}^{-1}$, the corresponding peak stress values of the Post-ECAP and Pre-ECAP samples reach 375 and 430 MPa, respectively. The difference in the values is not much.

Figure 3 reveals two things. On the one hand, the microstructure greatly influences the hot compressing deformation behavior of the Ti-6Al-4V alloy. Therefore, the Post-ECAP shows more obvious dynamic softening characteristics during the hot deformation process due to its finer grains and higher distortion energy. On the other hand, the Post-ECAP sample exhibits higher strength at 500 °C and lower flow stress at temperatures above 600 °C, promoting the plasticity-forming processes of the Ti-6Al-4V alloy.

**Figure 5:**
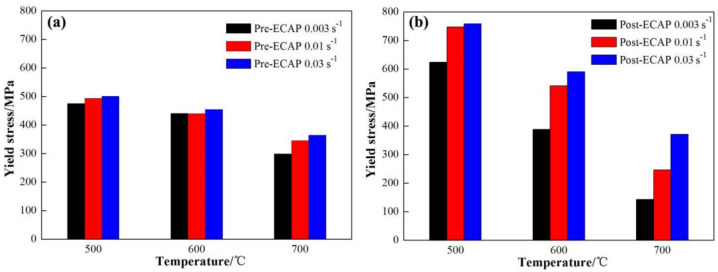
Variation of yield stress with temperature and strain rate: (**a**) Pre-ECAP; (**b**) Post-ECAP samples.

There is also a visible difference in the yields of the Pre-ECAP and Post-ECAP. The flow stress of Post-ECAP has slightly increased after the yield point; however, the yield characteristic of the Pre-ECAP is not obvious and exhibits significant work-hardening characteristics (Figure 3). The corresponding stress at the yield strain of 0.2% is uniformly defined as the yield stress to facilitate the analysis. Figure 5 shows that the yield stress of Pre-ECAP and Post-ECAP samples change with the deformation temperature and strain rate. The yield stress of Pre-ECAP is insensitive to the strain rate in the temperature range of 500 to 700 °C, with a slight change in values with increasing strain rate (from $\dot{\varepsilon }=\;0.003{\;s}^{-1}$ to $\dot{\varepsilon }=\;0.03{\;s}^{-1}$). In contrast, the yield stress of Post-ECAP samples increased significantly when strain increased from $\dot{\varepsilon }=\;0.003{\;s}^{-1}$ to $\dot{\varepsilon }=\;0.03{\;s}^{-1}$. Furthermore, the yield stress of Post-ECAP changes a lot with temperature change. The change in the yield stress values reveals that the ultra-fine grained Ti-6Al-4V alloy prepared by the multi-pass ECAP technique has a higher sensitivity toward deformation temperature and strain rate. Moreover, it also can be seen that the slope of post-ECAP flow curves increased with the increase of strain rate. On one hand, the yield flow stress increased with the increase of strain rate at the beginning of deformation. On the other hand, the high strain rate is beneficial to the dynamic recrystallization of Post-ECAP in the subsequent deformation, which lead to a rapid decrease of flow stress.

The changes in the deformation conditions have a great effect on the plastic processing property of Post-ECAP. Therefore, it is important to study the hot compression deformation behavior of the ultra-fine grained Ti-6Al-4V alloys prepared by the multi-pass ECAP technique to optimize the hot-working technology.

## 4. Discussions

### 4.1. Dynamic Softening Coefficient

**Figure 6:**
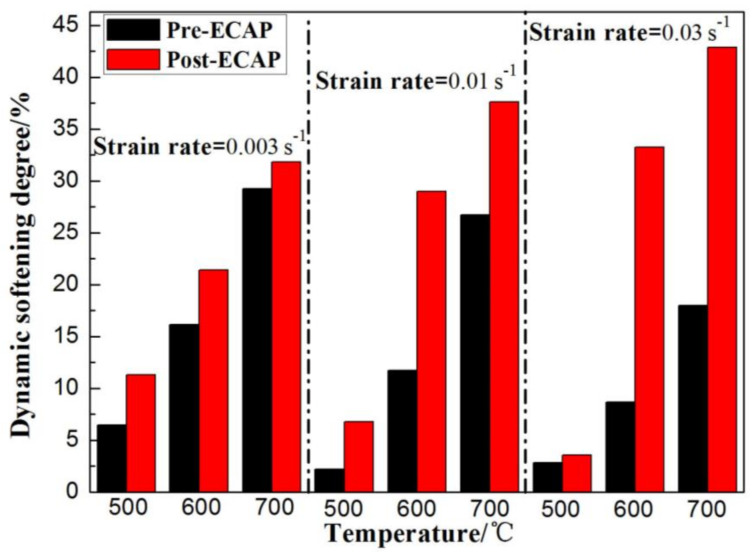
Dynamic softening coefficient of Ti-6Al-4V alloy during hot compression tests.

During the hot deformation process, the stress–strain curve can be divided into the elastic deformation stage, the work-hardening stage, and the dynamic softening stage. The dynamic softening step is a combination of the dynamic recovery and dynamic recrystallization processes. According to the stress–strain curves of the Pre-ECAP and Post-ECAP samples (Figure 3), the work-hardening effect in Post-ECAP is not significant. Still, the dynamic softening characteristic is more pronounced than Pre-ECAP. Furthermore, the dynamic recrystallization of Ti-6Al-4V became pronounced with increasing temperature and decreasing strain rate. Compared to Pre-ECAP, the dynamic recrystallization in Post-ECAP samples took place at lower temperature and higher strain rate. The dynamic softening coefficient *S_r_* of Ti-6Al-4V alloy at different deformation conditions can be calculated from Equation (1), as follows (Figure 6) [32]:

(1) $$S_{r}=\frac{\sigma_{p}-\sigma_{i}}{\sigma_{p}}\times 100\%\;\;$$

where $\sigma_{p}$ is the maximum stress, $\sigma_{i}$ is the stress at the end of the deformation process, i.e., the stress at a true strain value of 0.69. In general, the dynamic softening degree and percentage of dynamic recrystallization increase with increasing *S_r_*. As shown in Figure 6, the *S_r_* values of the Ti-6Al-4V alloy increase with increasing temperature, indicating that dynamic softening is promoted with increasing deformation temperature and decreasing work-hardening effects and deformation resistance. At 500 °C, the *S_r_* values of Pre-ECAP at a strain rate in the range of $\dot{\varepsilon }=0.003{\;s}^{-1}$ to $\dot{\varepsilon }=0.03{\;s}^{-1}$ are quite small, indicating that the dynamic recovery process is the major contributor in the dynamic softening behavior of Pre-ECAP. Dynamic recrystallization is almost absent during the deformation process. However, the *S_r_* values of the Post-ECAP samples increase with decreasing strain rate at 500 °C. Significantly dynamic softening was observed at 500 °C during the deformation process of Post-ECAP. The dynamic softening behavior becomes more prominent with decreasing strain rate at a lower temperature (500 °C). However, the trend in the variation of the *S_r_* values of the Pre-ECAP and Post-ECAP samples with increasing strain rate at deformation temperatures of 600 °C and 700 °C are different. The *S_r_* values of Pre-ECAP decreased with increasing strain rate, while the *S_r_* values of Post-ECAP increased with increasing strain rate. The trend is closely related to their microstructure, specifically the energy difference of distortion, which leads to the different dynamic softening mechanisms in Pre-ECAP and Post-ECAP samples. The large deformation seen in the Post-ECAP samples leads to high dislocation density and a large energy difference of distortion between grain boundaries [33]. This promotes the dynamic recrystallization at high strain rates due to the large driving force of recrystallization nucleation. However, the dislocation density of Pre-ECAP is low because of sample annealing (before deformation). The work-hardening effect was observed during the hot compression process. The dislocation density increased for the samples at this point. The driving forces required for dynamic recrystallization need to be accumulated during the deformation process. Therefore, the dynamic recovery process contributes the maximum during the dynamic softening of the deformation process of Pre-ECAP. The effect of dynamic recovery is less apparent at a lower strain rate.

### 4.2. Effect of Strain Rate on the Dynamic Softening Mechanism

**Figure 7:**
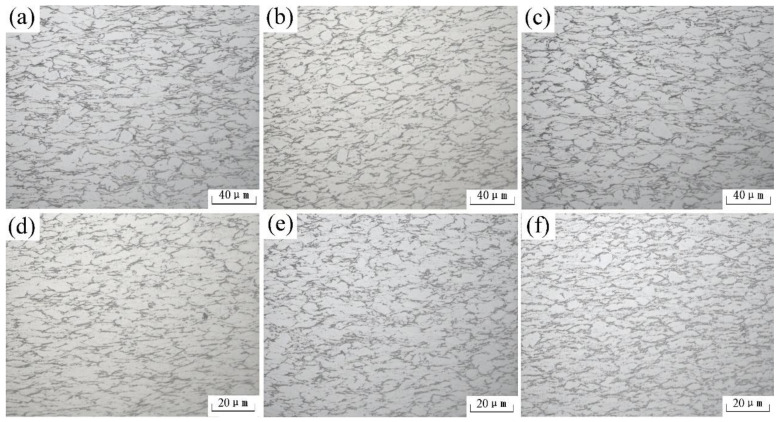
Evolution of microstructure of the Ti-6Al-4V alloy under different strain rates (500 °C). (**a**) Pre-ECAP, $\dot{\varepsilon }=0.003\;s^{-1}$; (**b**) Pre-ECAP, $\dot{\varepsilon }=0.001{\;s}^{-1}$; (**c**) Pre-ECAP, $\dot{\varepsilon }=0.03\;s^{-1}$; (**d**) Post-ECAP, $\dot{\varepsilon }=0.003{\;s}^{-1}$; (**e**) Post-ECAP, $\dot{\varepsilon }=0.01{\;s}^{-1}$; (**f**) Post-ECAP, $\dot{\varepsilon }=0.03\;s^{-1}$.

The dynamic softening mechanism can be studied by observing the changes in the microstructure of the materials after hot deformation. When only dynamic recovery takes place, the grains become elongated and distributed along the direction of deformation. A few subgrains may also be formed during the deformation process. However, many fine equiaxed recrystallized grains are formed if dynamic recrystallization occurs during the deformation process. On one hand, the grain size decreases due to dynamic recrystallization. On the other hand, the orientation of the grains is weakened because of the dynamic recrystallization process. The microstructures of the Pre-ECAP and Post-ECAP samples deformed at 500 °C under different strain rates (Figure 7).

As shown in Figure 7a–c, the strain rate has little effect on the microstructures of Pre-ECAP at a deformation temperature of 500 °C. The grain shape changed from equiaxed to long strip. Almost no recrystallization grain formed, indicating that dynamic recovery was the main dynamic softening mechanism when Pre-ECAP samples deformed at 500 °C. However, the grains aspect ratio in the Pre-ECAP samples increased with decreasing strain rate. Intracrystalline plastic deformation can be eliminated at a low strain rate leading to large deformation of grains [34]. This is mainly because the deformation material has enough time for dynamic recovery at a lower strain rate. The concentration stress caused by work hardening is released, leading to the decreased deformation resistance of the Pre-ECAP samples with decreasing strain rate. The microstructures of the Post-ECAP samples were deformed at 500 °C (Figure 7d–f). It can be seen that the proportion of dynamic recrystallization increases with increasing strain rate. The dislocation density of Post-ECAP samples is very high, and lots of subgrains are formed during the ECAP deformation process, indicating that the distortion energy of Post-ECAP was quite high and prone to dynamic recrystallization. Therefore, the dynamic recrystallization of Post-ECAP can rapidly occur even at low temperatures (500 °C) under high strain rates ($\dot{\varepsilon }=0.03\;s^{-1}$). The higher strain rate was beneficial for the accumulation of the distortion energy difference and the rapid dynamic recrystallization. This resulted in super-refined grains.

### 4.3. Effect of Temperature on the Dynamic Softening Mechanism

**Figure 8:**
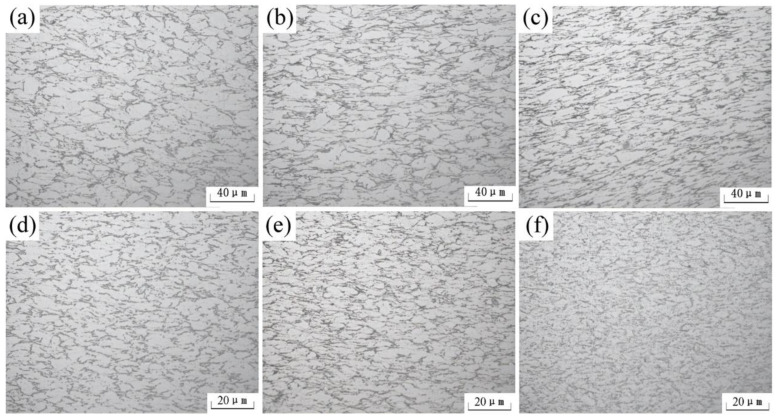
Evolution of microstructure of the Ti-6Al-4V alloy at different temperatures ($\dot{\varepsilon }=0.03{\;s}^{-1}$). (**a**) Pre-ECAP, 500 °C; (**b**) Pre-ECAP, 600 °C; (**c**) Pre-ECAP, 700 °C; (**d**) Post-ECAP, 500 °C; (**e**) Post-ECAP, 600 °C; (**f**) Post-ECAP, 700 °C.

In metals, the thermal motion of atoms and the mobility of dislocations is enhanced with increasing temperature. Dynamic recovery and dynamic recrystallization are also facilitated. The microstructures of Pre-ECAP and Post-ECAP deform under a high strain rate of $\dot{\varepsilon }=0.03\;s^{-1}$ (Figure 8). It can be seen that the dynamic recrystallization behavior is enhanced as the temperature increased for both Pre-ECAP and Post-ECAP. Dynamic recrystallization was not observed for Pre-ECAP samples at a deformation temperature of 500 °C. However, a small amount of fine recrystallized grains was observed at 600 °C. When the deformation temperature rose to 700 °C, the dynamic softening effect was further enhanced, and the dynamic recrystallization process became more apparent, resulting in refined grains. A comparison of the microstructures of Pre-ECAP and Post-ECAP samples, which deformed under the same conditions, shows that the dynamic recrystallization behavior in Post-ECAP was more prominent. Finer grains were produced after deformation. As shown in Figure 8f, almost fully dynamic recrystallization had taken place during the deformation process of Post-ECAP at 700 °C. Fine equiaxial grains were produced during the process.

The results revealed that dynamic softening behavior is different in Pre-ECAP and Post-ECAP samples under the same conditions. The primary softening mechanism of Pre-ECAP is dynamic recovery at 500 °C. Dynamic recrystallization was almost absent. Incomplete recrystallization occurred in Post-ECAP during the deformation process at 500 °C when the strain rate was high. This indicated that the dislocation density was very high, and the work-hardening capacity increased continuously. The stress of Post-ECAP is higher than Pre-ECAP at 500 °C (Figure 3). However, with an increase in the deformation temperature, the dynamic recrystallization behavior becomes more prominent and facilitates stress reduction. Moreover, the dislocation density and grain size decreased due to the increase in the volume fraction of dynamic recrystallization. The softening capacity of Pre-ECAP was lower than Post-ECAP due to incomplete dynamic recrystallization. Therefore, the stress of Post-ECAP significantly decreased (values below those of Pre-ECAP) at 600 and 700 °C under the same strain rate. Enhancement of the dynamic softening effect and further refinement of grains were responsible for the observed effects.

### 4.4. Effect of Initial Microstructure on the Dynamic Recrystallization Behavior

**Figure 9:**
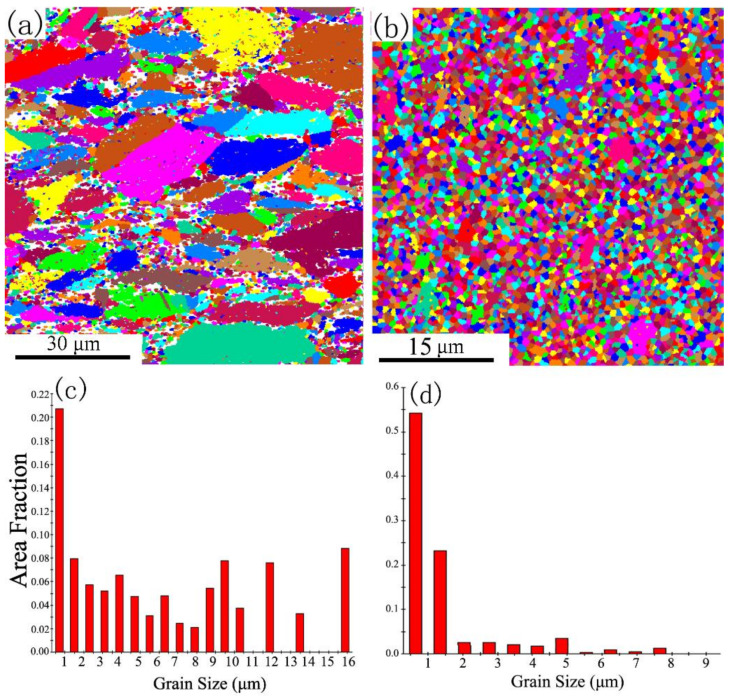
Grain size distribution of Ti–6Al–4V alloy after hot compression (700 °C, $\dot{\varepsilon }=0.01{\;s}^{-1}$). (**a**) Electron back-scattered diffraction (EBSD) map of Pre-ECAP; (**b**) EBSD map of Post-ECAP; (**c**) Grain size distribution of Pre-ECAP; (**d**) Grain size distribution of Post-ECAP.

The EBSD figures of Pre-ECAP and Post-ECAP deformed samples at 700 °C under a strain rate of $\dot{\varepsilon }=0.01\;s^{-1}$ are shown in Figure 9. The original large grains were present in the deformation microstructure of the Pre-ECAP, but the grain shape was elongated along the perpendicular direction of the compression deformation. Furthermore, fine recrystallization grains appeared around the original big grains. However, the microstructure of Post-ECAP samples was uniform and refined due to fully dynamic recrystallization during the deformation process. According to the grain size distribution of Post-ECAP samples, the proportion of grains with sizes of less than 1 µm is more than 50%. Studies have shown that the ultra-fine grain materials with uniform and stable microstructure can be prepared by a method combining large plastic deformation and deformation heat treatment [35,36]. The grains of Post-ECAP samples had been refined by multi-pass ECAP [26]. The microstructure of the Post-ECAP sample is not stable and has lots of subgrains and twins [29]. However, the unstable microstructure transformed into a uniform and stable microstructure with fine equiaxed grains following further compression, which would be beneficial for the microstructure and mechanical properties of Ti-6Al-4V alloy.

**Figure 10:**
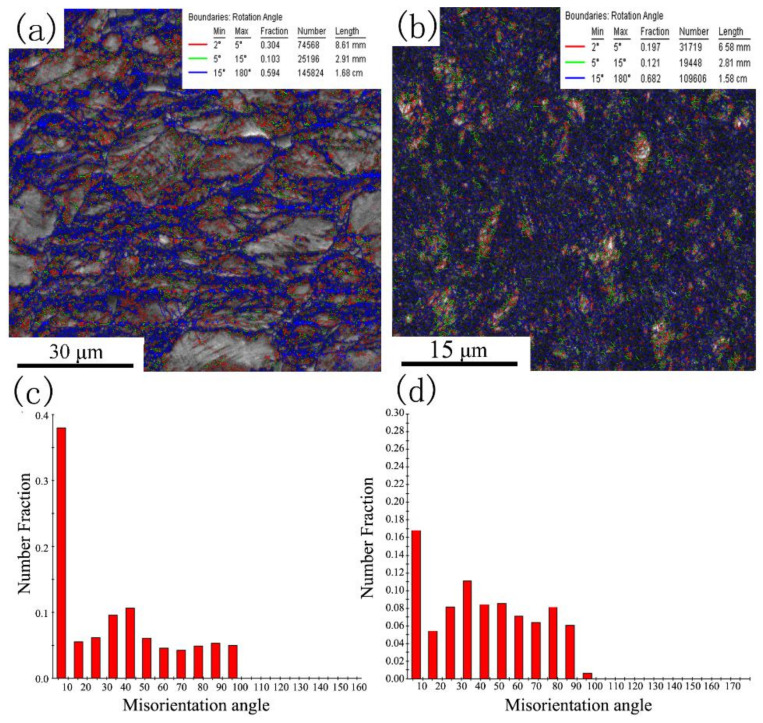
Grain boundary misorientation in Ti-6Al-4V alloy (700 °C, $\dot{\varepsilon }=0.01\;s^{-1}$). (**a**) Pre-ECAP; (**b**) Post-ECAP; (**c**) Misorientation angle distribution in Pre-ECAP; (**d**) Misorientation angle distribution in Post-ECAP.

The misorientation angle distributions of Pre-ECAP and Post-ECAP samples deformed at 700 °C at a strain rate of $\dot{\varepsilon }=0.01\;s^{-1}$ are shown in Figure 10. There were significant amounts of subgrain structures and a high proportion of low-angle grain boundaries in the initial microstructure of the Post-ECAP samples. However, the proportion of low-angle grain boundaries in Post-ECAP significantly decreased (lower than Pre-ECAP) after compression, indicating that fully dynamic recrystallization occurred during the hot compression deformation process of Post-ECAP. The increasing high-angle grain boundaries and decreasing low-angle grain boundaries caused by the dynamic recrystallization process were the main reasons behind the improvement of microstructure stability. In contrast, the proportion of low-angle grain boundaries in the Pre-ECAP samples was still high. Some substructures with low-angle grain boundaries came into being due to the accumulation of the dislocations during the Pre-ECAP deformation process. However, the energy difference of distortion between the grain boundaries caused by the dislocation motion was still not adequate for dynamic recrystallization. Hence, unstable substructures with low-angle grain boundaries were present.

**Figure 11:**
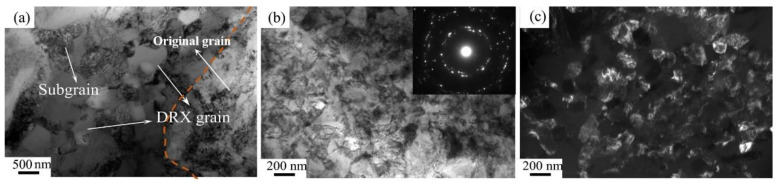
TEM microstructure of Ti-6Al-4V compressed at 600 °C and at $\dot{\varepsilon }=0.01\;s^{-1}$. (**a**) Pre-ECAP; (**b**) Post-ECAP; (**c**) Dark field of Post-ECAP.

Figure 11 shows the TEM microstructures of Pre-ECAP and Post-ECAP deformed at 700 °C under a strain rate $\dot{\varepsilon }=0.01\;s^{-1}$. For Pre-ECAP, the dynamic recrystallization grains in a size range of 200–300 nm were detected between the original grains. Moreover, subgrains with high dislocation density were detected around the recrystallization grains. However, as Figure 11b,c show, the microstructure of the deformed Post-ECAP sample is more uniform, and the sizes of the recrystallization grains are in the range of 100–200 nm, which is much smaller than that of Pre-ECAP. The regular ringed SAD pattern indicates that its microstructure is homogeneous and stable.

The results from the EBSD and TEM experiments revealed that the volume fraction of dynamic recrystallization of the Post-ECAP sample is higher than that of the Pre-ECAP sample under the same hot deformation conditions. The dynamic softening behavior is more pronounced, which results in a dramatic decrease in the flow stress during the hot deformation process. Ultra-fine grained Ti-6Al-4V alloy with a homogeneous and stable microstructure is obtained by the ECAP severe plastic deformation method followed by the hot compression technique. Thus, a new technique for the preparation of high-performance titanium alloy is developed. The fine, uniform, and the equiaxed grain structure is obtained using the hot plastic deformation technique of fabricating the Ti-6Al-4V alloy. This is also the main reason behind the constant decrease of the flow stress of Post-ECAP samples at 700 °C.

## 5. Conclusions

(1) The hot deformation behavior and microstructural evolution of Ti-6Al-4V alloy with two different initial microstructures were evaluated using a Gleeble simulator, OM, EBSD, and TEM techniques. The following conclusions could be drawn from this investigation:

(2) The stress of Post-ECAP is higher than Pre-ECAP overall strain rates at 500 °C. The stress decreased rapidly with increasing temperature and became lower than Pre-ECAP at 600 and 700 °C (especially at low strain rate).

(3) Post-ECAP exhibits significant dynamic softening behavior compared to Pre-ECAP. The dynamic recovery process is the main dynamic softening mechanism in Pre-ECAP. Dynamic recrystallization is more likely to occur in Post-ECAP due to its higher dislocation mobility and distortion energy difference, indicating that the thermoforming capacity of Post-ECAP is better than Pre-ECAP at temperatures higher than 600 °C.

Ultra-fine-grained Ti-6Al-4V alloy with a homogeneous and stable microstructure can be prepared by the ECAP severe plastic deformation and the hot compression technique, providing a new method for preparing the high-performance titanium alloys.

## Figures and Tables

**Table 1:** Deformation temperature and strain rates for the isothermal compression tests.

Materials	Conditions	
Pre-ECAP	Temperature	500 °C, 600 °C, 700 °C
	Strain rate	0.003 s^−1^, 0.01 s^−1^, 0.03 s^−1^
Post-ECAP	Temperature	500 °C, 600 °C, 700 °C
	Strain rate	0.003 s^−1^, 0.01 s^−1^, 0.03 s^−1^

## Data Availability

Data available in a publicly accessible repository.
